# Regulation of the Switchable Luminescence of Tridentate Platinum(II) Complexes by Photoisomerization

**DOI:** 10.3389/fchem.2020.622256

**Published:** 2021-02-05

**Authors:** Yongguang Li, Yuexuan Fei, Hongcheng Sun, Shuangjiang Yu, Junqiu Liu

**Affiliations:** College of Material, Chemistry and Chemical Engineering, Hangzhou Normal University, Hangzhou, China

**Keywords:** luminescence, platinum(II), photoisomerization, dual-emission, azobenzene (abz)

## Abstract

Organoplatinum (II) complexes are promising candidates for the construction of smart supramolecular materials due to their unique flat structures. This accompanied by intriguing luminescent properties, prompts the molecules to aggregate after external stimuli. Nevertheless, the utilization of photo-responsive subunits to modulate their assemble behaviors and functions are still rarely explored. In this work, azobenzene (azo)-appended tridentate platinum (II) complexes with different linkers have been designed and synthesized. The intermolecular hydrogen bonding, *π*-π stacking, and metal-metal interactions were finely controlled through the tiny alteration of the linkers, which was found to play a vital role in self-assembly, and photophysical and photoisomerization properties. Some of them exhibited dual emission bands originating from metal-perturbed triplet intraligand (^3^IL) and metal-metal to ligand charge transfer (^3^MMLCT) excited states due to the different intermolecular interactions. Based on this, the manipulation of switchable luminescence as well as the controllable morphologies have been realized by photoisomerization.

## Introduction

Coordinated complexes of d^8^ transition metals with various *π*-conjugated ligands are prone to stack and form aggregates with fascinating luminescence characteristics (Zhao et al., 2011; Yam et al., 2015; Zhang et al., 2018a; Li et al., 2019; Li et al., 2020a). In view of their sensitive and changeable photophysical behaviors, they are promising candidates for application in the fields of chemosensors and bio-imaging (Xiang et al., 2013; Zhang et al., 2018b; Li et al., 2020b; He et al., 2020). So far, abundant luminescent materials based on planar-structured transition metal complexes have been accurately tailored by the combination of molecular structures and intermolecular interactions (Gao et al., 2018; Sinn et al., 2018; Kang et al., 2019; Sun et al., 2019; Yang et al., 2020). For instance, platinum (II) tetraphenylporphyrin polymers have been employed as dual-emissive materials (Wang et al., 2018). They originated from chromophore platinum (II) and a clustering-triggered polymer, respectively. The dual-emissive materials are used as ratiometric biological sensors to trace hypoxia *in vivo*. We also used the cyclometalated platinum (II) and rigid aromatic alkynyl groups as the building blocks to construct a luminescent molecular hinge (Ai et al., 2019). The motions of the molecular hinge could be driven by solvent and temperature. This could be conveniently detected by drastic emissive color changes. Therefore, the dynamic control and regulation of the molecular aggregates is a vital strategy to construct smart luminescent materials (Xiao et al., 2020a; Xiao, et al., 2020b).

**FIGURE 1:**
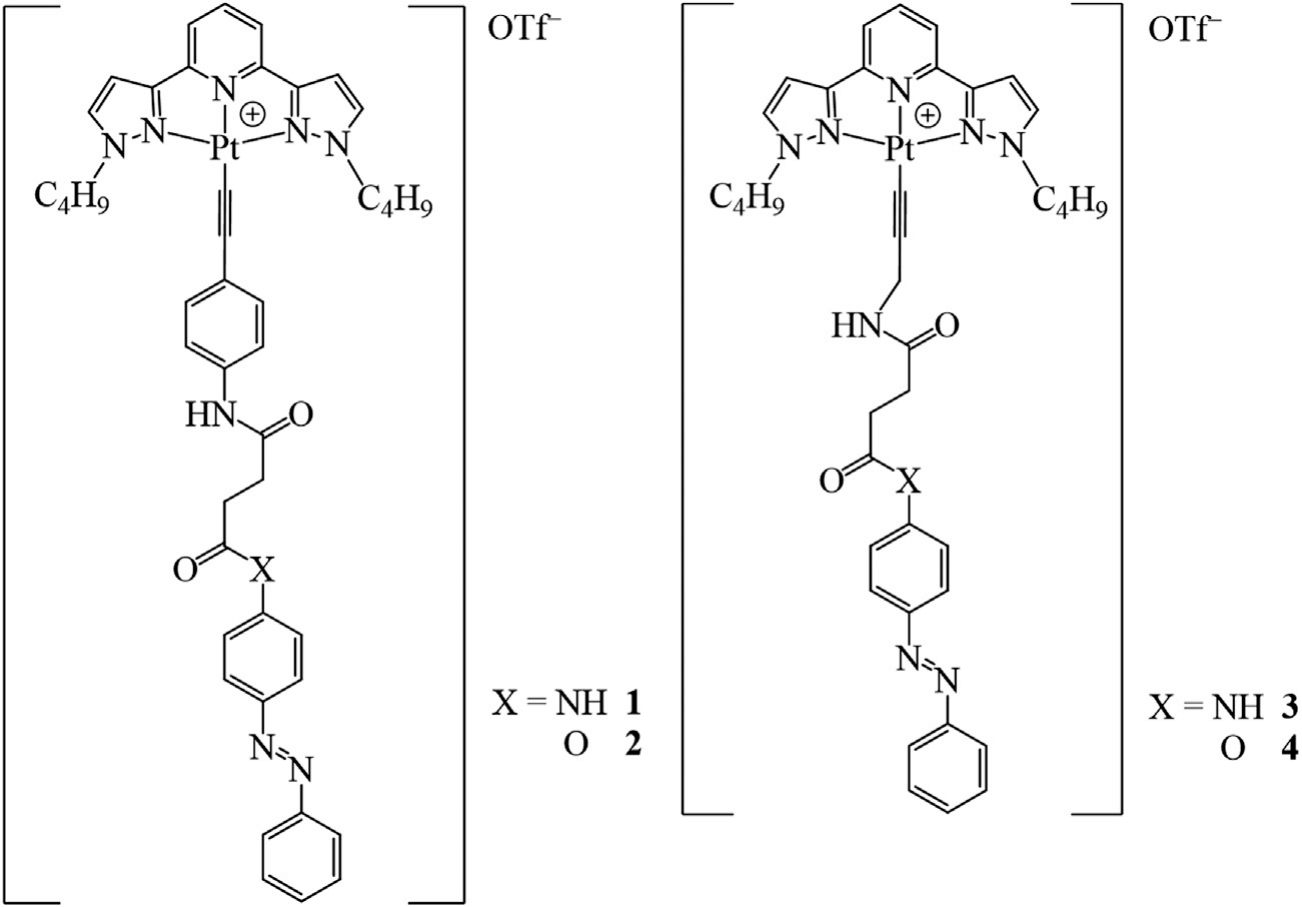
Molecular structures of complexes **1**–**4**.

The aggregates from coordinated transition metal complexes are susceptible to external stimuli, such as solvent, temperature, pressure, pH value, gas, and chemicals due to labile and weak noncovalent interactions (Chang et al., 2012; Au et al., 2013; Wenger, 2013; Zhao et al., 2017; Cretu et al., 2018; Dalapati et al., 2019; Shigeta et al., 2019). The electronic distribution also changes upon the modulation of the molecular assembly (Xiao et al., 2019), resulting in the emergency of diversified photophysical properties of the luminescent molecules in solution, aggregate, solid, and glass states. One of the examples is the bipyridine platinum (II) dichloride complex which is well-known for the red and yellow dimorphism in the solid state due to the different separation of metal-metal distances depending on recrystallized conditions (Herber et al., 1994). Among the various external stimuli, illumination is one of the ideal choices because no chemical wastes are produced which may alter the microenvironment and subsequently influence the intermolecular interactions and reversibility of the assembly properties. Photo-responsive groups are introduced into supramolecular self-assembly systems. Under light irradiation, photoisomerization of the photo-responsive units occurs accompanied by morphological transitions (Chen et al., 2010; Tanaka et al., 2012; Lin et al., 2020). Cyanostilbene modified with dimethylaniline was found to self-assemble into vesicles and fibrous morphologies depending on solvents. Their morphologies could be changed with enhanced emission triggered by the photoisomerization of cyanostilbene (Yang et al., 2016). Photo-switchable functional groups such as azo, dithienylethene, spiropyran, spirooxazine, and so forth are introduced to the transition metal complex systems (Li et al., 2011; Deibel et al., 2013; Fihey et al., 2015; Li et al., 2015; Perez-Miqueo et al., 2015; Ko and Yam, 2018; Bhattacharyya et al., 2020). For example, the emission intensity of the *cis* isomer of azo-linked diporphyrin zinc(II) complexes was much weaker than that of the *trans* isomer because the intramolecular electron transfer occurred from the electron-rich porphyrin zinc(II) complex to the electron-deficient porphyrin zinc(II) moiety (Tsuchiya, 1999). In addition, the photoisomerization can be sensitized by transition platinum (II) metal complexes when used as triplet donors as well as enhanced spin-orbit coupling (Li et al., 2011; Ko and Yam, 2018). Although some works related to the fluorophores of platinum (II) modified with photoisomeric azo groups and their photophysical properties have been reported (Yutaka et al., 2002; Sakamoto et al., 2005; Sakamoto et al., 2009; Moustafa et al., 2012), to the best of our knowledge, the investigation focusing on the switching of the self-assembled luminescent metal complexes still requires exploration. Inspired by this, we designed and synthesized a series of azo-appended tridentate platinum (II) complexes with different linkers (Figure 1). They exhibited different luminescent properties due to a tiny alteration of the connected units and showed switchable emission behaviors with morphologic changes triggered by the photoisomerization of azo.

## Results and Discussion

**SCHEME 1:**
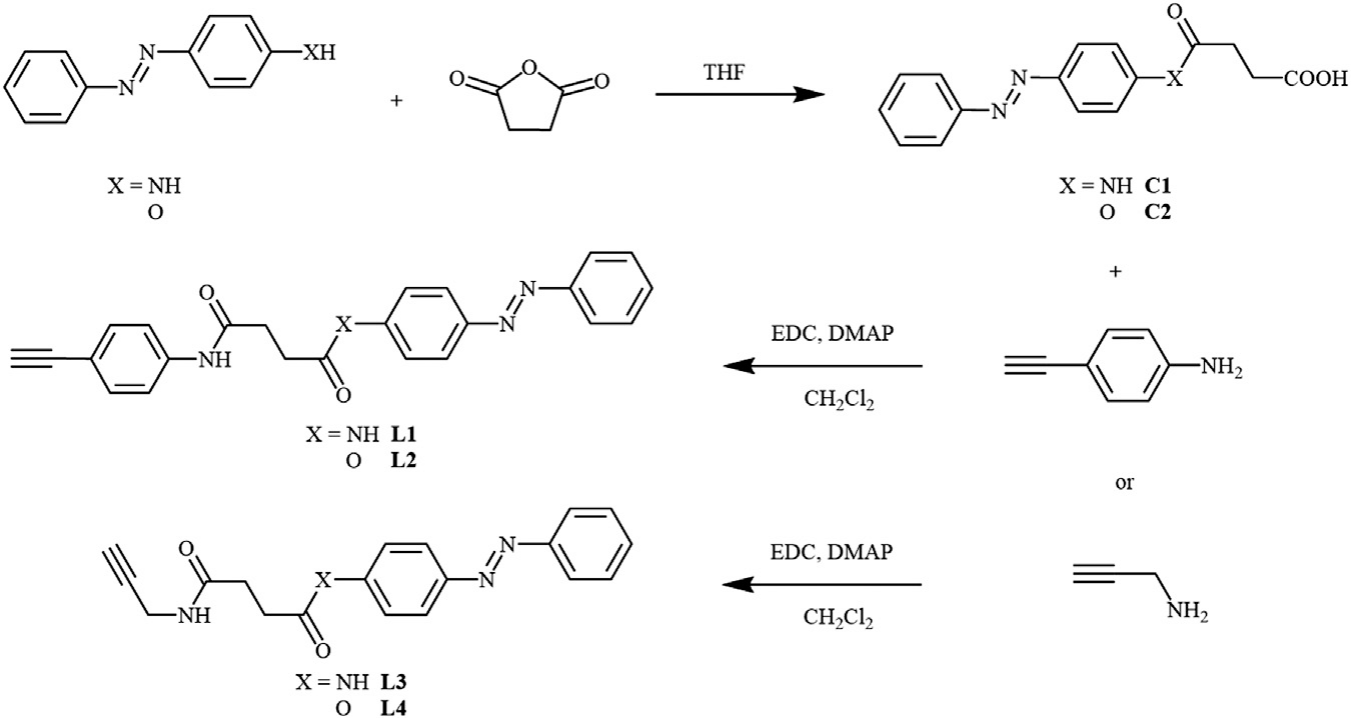
Synthetic routes of ligands **L1**–**L4**.

**SCHEME 2:**
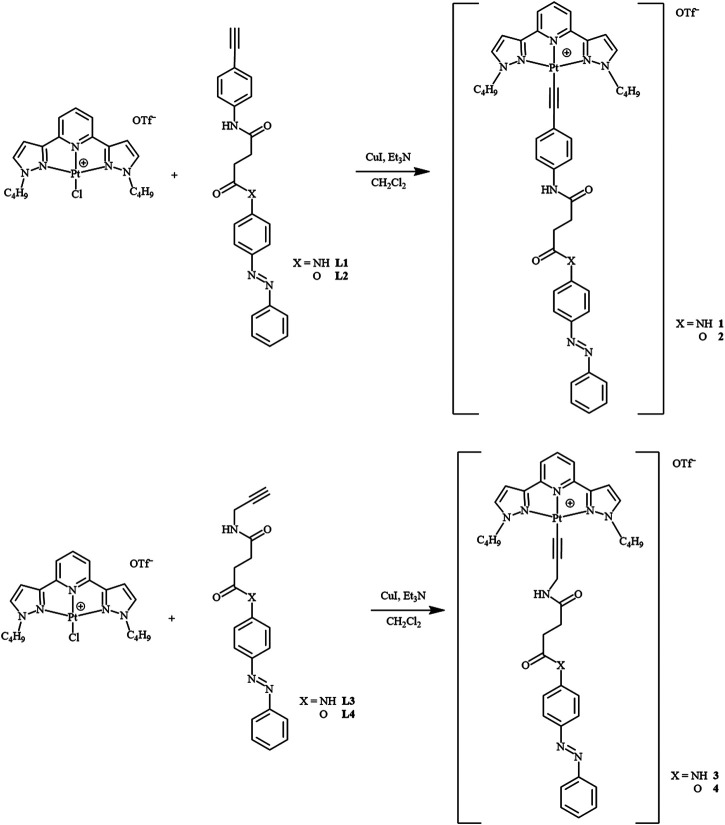
Synthetic routes of complexes **1**–**4**.

The azo alkynylplatinum (II) complexes **1**–**4** (Figure 1) were synthesized by the reaction of chloroplatinum (II) precursors with the respective azo alkynes **L1**–**L4** [(*E*)-N^1^-(4-ethynylphenyl)-N^4^-(4-(phenyldiazenyl)phenyl)succinamide (**L1**) (*E*)-4-(phenyldiazenyl)phenyl 4-((4-ethynylphenyl)amino)-4-oxobutanoate (**L2**) (*E*)-N^1^-(4-(phenyldiazenyl)phenyl)-N^4^-(prop-2-yn-1-yl)succinamide (**L3**), and (*E*)-4-(phenyldiazenyl)phenyl 4-oxo-4-(prop-2-yn-1-ylamino)butanoate (**L4**)] according to the previously reported method (Li et al., 2016). The reaction of 4-ethynylaniline or prop-2-yn-1-amine with (*E*)-4-oxo-4-((4-(phenyldiazenyl)phenyl)amino)butanoic acid (**C1**), or (*E*)-4-oxo-4-(4-(phenyldiazenyl)phenoxy)butanoic acid (**C2**) in the presence of 1-ethyl-3-(3-dimethylaminopropyl) carbodiimide hydrochloride (EDC) and 4-(dimethylamino)pyridine (DMAP) yielded the respective desired ligands in good yield. The compounds **C1** and **C2** were prepared by an acylamidation reaction of (*E*)-4-(phenyldiazenyl)aniline, or (*E*)-4-(phenyldiazenyl)phenol with succinic anhydride. All the intermediates were characterized by ^1^H NMR spectroscopy. The final complexes were characterized by ^1^H NMR, FAB-mass spectrometry, and elemental analysis. The synthetic routes (Schemes 1 and 2) and details of the corresponding ligands and final complexes are described in the Materials and Methods section.

**FIGURE 2:**
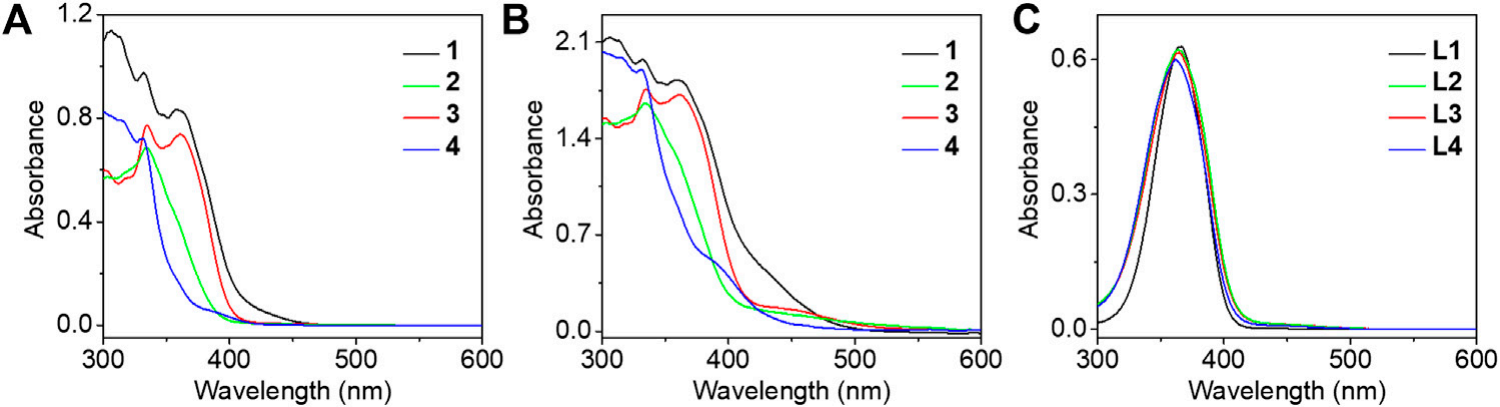
The electronic absorption spectra of complexes **1**–**4** were studied in DMSO solution at a concentration of 4.62 × 10^–5^ **(A)** and 8.53 × 10^–4^ mol/L **(B)**, and **(C)** the electronic absorption spectra of corresponding ligands **L1**–**L4** in DMSO solution at a concentration of 3.87 × 10^–5^ mol/L.

**FIGURE 3:**
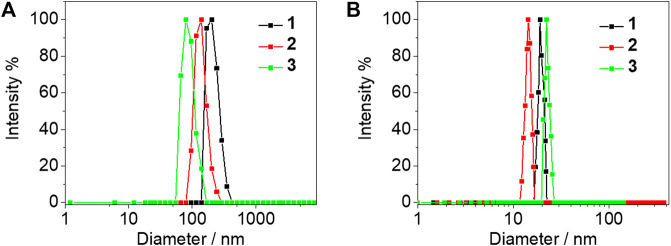
DLS experiments of complexes **1**–**3** before **(A)** and after **(B)** 365 nm UV light irradiation for 2 min in DMSO (8.53 × 10^–4^ mol/L) at 25°C. The number-averaged hydrodynamic diameters before and after 365 nm UV light irradiation were found to be 201.5 (**1**), 118.0 (**2**), and 81.7 (**3**) nm, and 19.0 (**1**), 14.4 (**2**), and 22.3 (**3**) nm, respectively.

The electronic absorption spectra of complexes **1**–**4** were studied in DMSO solution as shown in Figure 2A. Strong absorption bands at 300–365 nm and moderately intense absorption shoulders around 390 nm were observed. In comparison with the electronic absorption spectra of the alkynyl ligands **L1**–**L4** (Figure 2C) and previously reported work (Li et al., 2016), the absorption bands before 350 nm were assigned to the π→π* intraligand (IL) transition of the 2,6-bis(1-alkylpyrazol-3-yl)pyridine tridentate ligand, whereas the absorption bands and shoulders around 390 nm were tentatively assigned as the π→π* transition of azo moiety mixed with the dπ(Pt)→π*(2,6-bis(1-alkylpyrazol-3-yl)pyridine) metal-to-ligand charge transfer (MLCT) transition. As the concentration increased to 8.53 × 10^–4^ mol/L, a new absorption band appeared at 450 nm of complexes **1**–**3**, typically originating from the metal-metal to ligand charge transfer (MMLCT) transition which is attributed to the formation of intermolecular *π*-π and metal-metal interactions (Figure 2B), but no obvious MMLCT absorption band was measured for complex **4**. In the concentrated DMSO solution, the dynamic light scattering (DLS) results also revealed that complexes **1**–**3** aggregated with the observation of hydrodynamic diameters (D_h_) of 201.5, 138.9, and 79.5 nm, respectively. However, no aggregated phenomenon was observed for complex **4** in the same condition (Figure 3A). The results demonstrated that both complexes **1** and **3** with double amide groups have a serious tendency to aggregate through hydrogen bonding, *π*-π, and metal-metal interactions. Although only one amide group was introduced to complex **2** compared with complexes **1** and **3**, the reason for the formation of aggregates was probably due to the extra *π*-π stacking interaction from the phenylethynyl groups which was observed in our previously reported platinum (II) complexes with the formation of metallogels (Ai et al., 2016). According to the electronic absorption and DLS results, complex **4** with only one propargyl amide group could not be assembled in the concentrated DMSO solution. This revealed that the designation of molecular structures is of great importance for the construction of functional materials.

**FIGURE 4:**
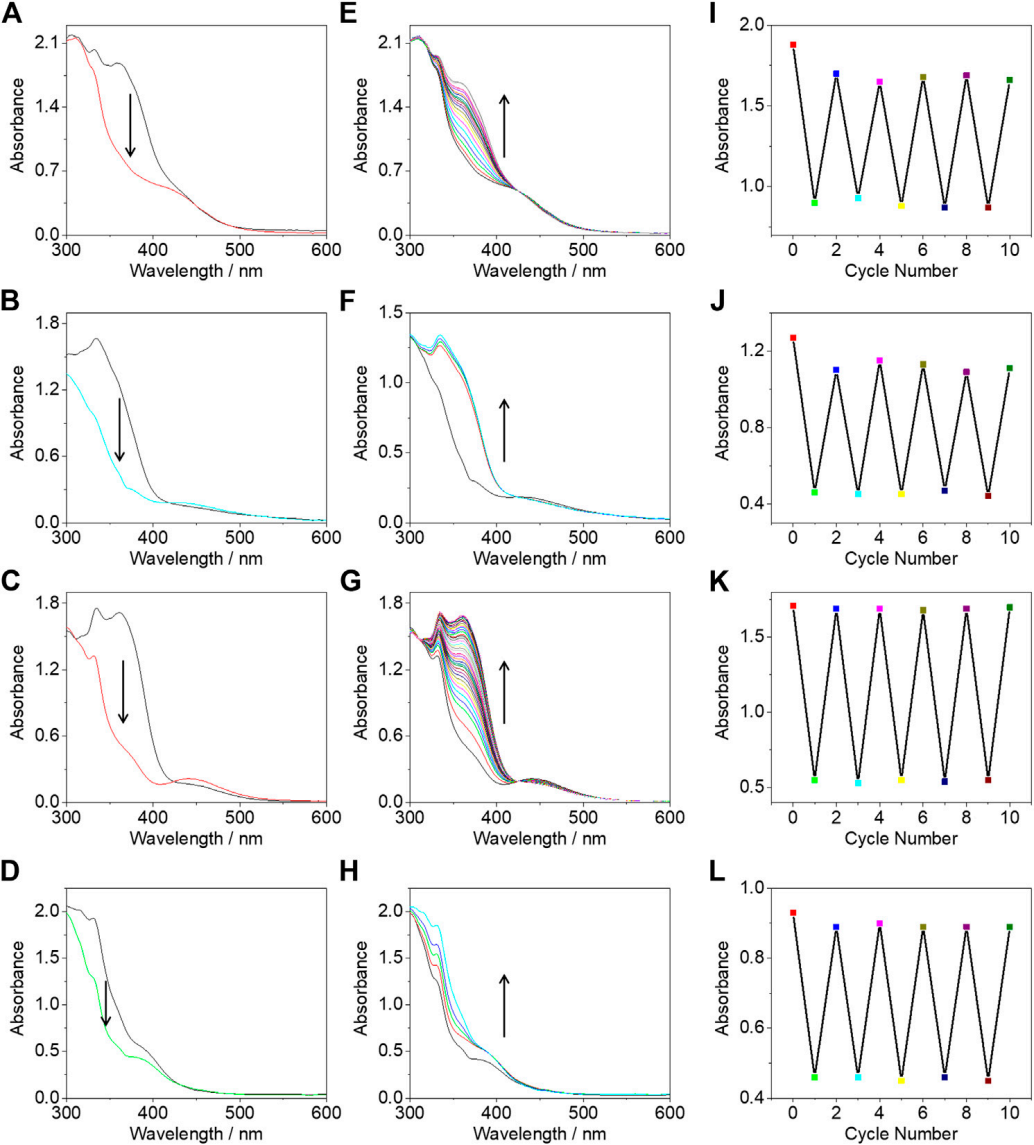
The electronic absorption spectral change of complexes **1 (A)**, **2 (B)**, **3 (C)**, and **4 (D)** under 356 nm UV light irradiation for 2 min. Electronic absorption spectral change of complexes **1 (E)**, **2 (F)**, **3 (G)**, and **4 (H)** under 450 nm visible light irradiation for 15 min. Absorption intensity at 361 nm for complexes **1 (I)**, **2 (J)**, **3 (K)**, and **4 (L)** under alternate irradiations with 365 and 450 nm light. All the experiments were performed at a concentration of 8.53 × 10^–4^ mol/L in deaerated DMSO.

The photoisomerization properties were studied through the electronic absorption spectra which could be conveniently monitored by an electronic absorption spectrometer. Upon irradiation under 365-nm UV light for 2 min, the absorption bands of complexes **1**–**3** at *ca.* 361 nm of π→π* transition of azo immediately decreased with the increase of the absorption band around 445 nm, which was overlapped with the MMLCT absorption bands arising from the intermolecular *π*-π and metal-metal interactions (Figures 4A–C). This increased absorption band was assigned as the n→π* transition of azo. Complex **4** also showed *trans*-*cis* isomerization under 365 nm UV light irradiation in concentrated solution (Figure 4D). The reversible *cis* to *trans* isomerization could also be realized under visible light irradiation with the increase of the 361 nm absorption band and the decrease of the 445 nm absorption band (Figures 4E–H). There was no drastic intensity changes for the 445 nm absorption band due to the spin forbidden n→π* transition as well as the offset of the increased intensity of the adsorption band of MMLCT transition associated with the formation of *π*-π and metal-metal interactions. The possible reasons for the incomplete recovery of the initial state of the *trans* isomer include; i) the system achieved a photo-stationary state and ii) the photo-responsive azo group was not stable enough and partially photodegraded. After five cycles (Figures 4I–L), there was no obvious decrease of the 361 nm absorption intensity indicating that the photo-stationary state plays a major role.

**FIGURE 5:**
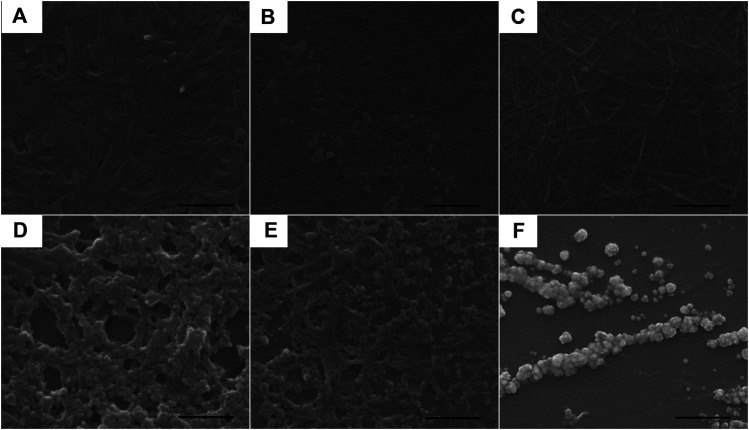
SEM images of the self-assembled aggregates prepared from the DMSO solutions of complexes **1**–**3** (8.53 × 10^–4^ mol/L) **(A**–**C)** before and **(D**–**F)** after exposure to 365 nm UV light for 2 min, respectively. The scale bar is 500 nm. Samples were prepared by dropping the respective solutions onto a silica wafer and the solvents were allowed to evaporate at room temperature.

Since the electronic absorption and DLS experiments of complexes **1**–**3** revealed that they were inclined to aggregate, their morphologies were further studied using scanning electron microscopy (SEM). The samples before and after UV light irradiation were dropped onto a silicon wafer, respectively and then removed from the solvents to obtain their SEM morphologies. As shown in Figure 5, before UV light irradiation, the SEM picture illustrated that the regulated nanorod with an average size of 400 nm in length and 45 nm in width was formed in complex **1**; continuous nanostructures were assembled in complex **2;** and nanofibers were observed in complex **3**. After UV irradiation for 2 min, aggregated nanospheres were observed for complexes **1**–**3** (Figures 5D–F). DLS results revealed distinct hydrodynamic diameter changes before and after photoisomerization as described in Figure 3. For the initial *trans* isomer, the phenyl groups of the azo moieties were coplanar. The complexes were able to align into nanofibers undergoing the noncovalent driving force including hydrogen bonding, *π*-π, and metal-metal interactions. However, the *cis* isomer with phenyl subunits twisting out-of-plane led to large steric hindrance. During the photoisomerization process, the noncovalent intermolecular interactions were disrupted due to the conversion of the coplanar *trans* isomer to a noncoplanar *cis* isomer, resulting in the morphological change from nanorods or nanofibers to nanospheres.

**FIGURE 6:**
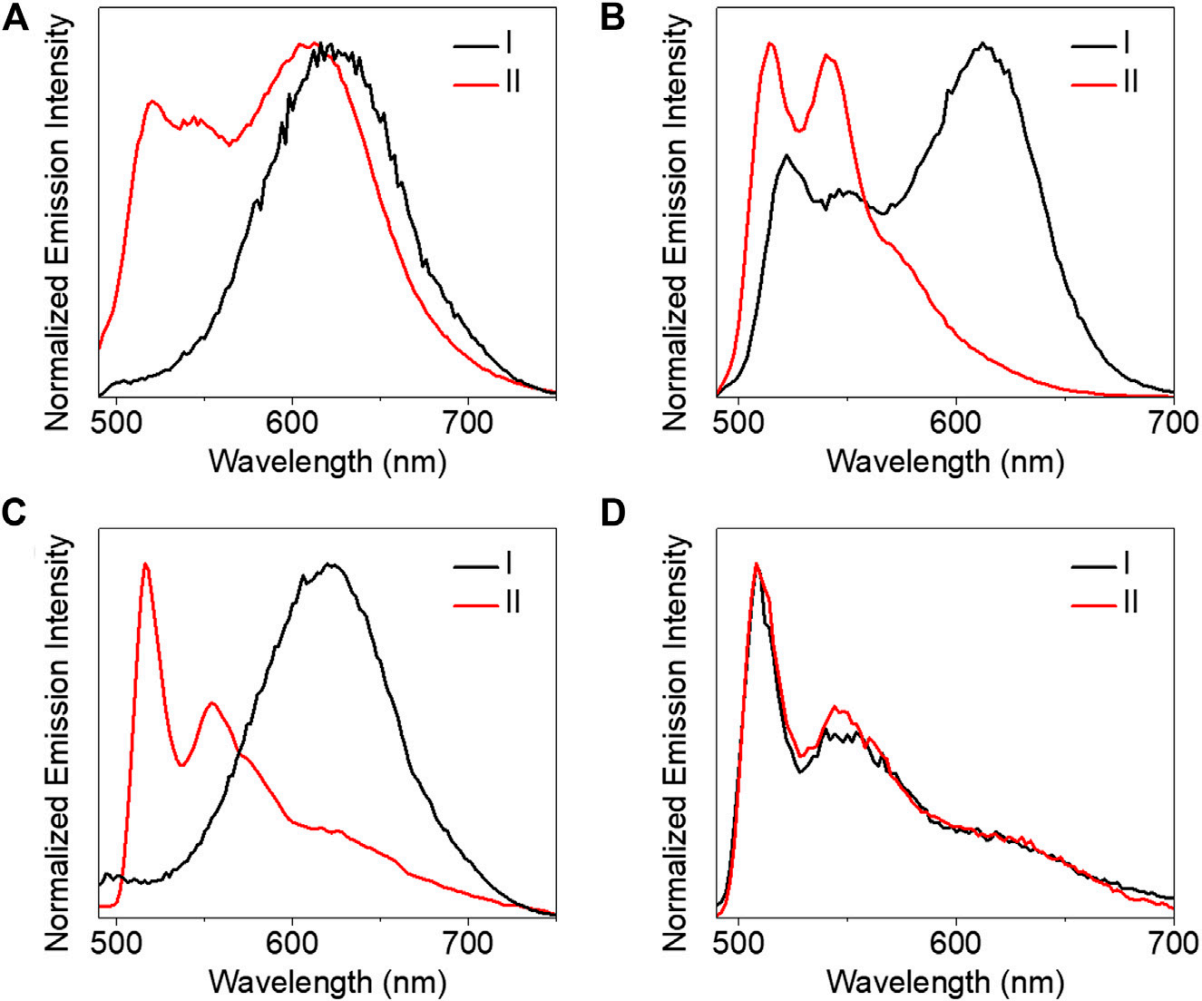
Emission spectra **(A**–**D)** of complexes **1**–**4** (8.53 × 10^–4^ mol/L) in MeTHF at 77 K (I) before and (II) after exposure to UV light (365 nm) for 5 min, respectively.

All the complexes were not emissive in both diluted and concentrated DMSO at room temperature. This is probably due to the introduction of the 2,6-bis(1-alkylpyrazol-3-yl)pyridine ligand. We will further investigate phosphorescence behaviors with platinum (II) coordinated to other ligands in our future works. Subsequently, we investigated their luminescent properties in 2-methyltetrahydrofuran (MeTHF) at 77 K. In the initial *trans* isomer state, both complexes **1** and **3** showed broad structureless emission bands around 620 nm (Figures 6A,C). Besides a broad structureless emission band at 612 nm, a well-resolved vibronic-structured and high emission energy band was also observed for complex **2** (Figure 6B). However, only a vibronic-structured emission band was observed for complex **4** (Figure 6D). The vibrational progression spacings of the well-resolved vibronic-structured emission were calculated in the range of 1,115–1,330 cm^−1^ which were characteristic of the aromatic C=C and C=N vibrational modes. So these high energy and vibronic-structured emission bands were assigned to the metal-perturbed π→π* ^3^IL excited state of the 2,6-bis(1-alkylpyrazol-3-yl)pyridine tridentate ligands mixed with some dπ(Pt)→π*(2,6-bis(1-alkylpyrazol-3-yl)pyridine) ^3^MLCT character. The low energy and structureless emission bands were tentatively acknowledged to originate from the ^3^MMLCT excited state. The observation of the ^3^MMLCT emission also indicated the formation of *π*-π and metal-metal interactions for both **1** and **3**. The appearance of a dual emission and vibronic-structured emission bands for complexes **2** and **4,** respectively, suggested that the noncovalent intermolecular interactions decreased.

Under 365 nm UV light irradiation, the azo moiety changed from the *trans* to *cis* isomer state. It is noteworthy that complex **1** showed dual emission bands including the appearance of a well-resolved vibronic-structured emission band along with the lower-energy and broad structureless emission band even after irradiation for 1 h. The result indicates that the aggregates were not completely destroyed after 365 nm UV irradiation. Only vibronic-structured emission bands were observed for complexes **2**–**4** which revealed a complete conversion of the *cis* isomer. The *cis* isomer always featured out-of-plane twisting of phenyl subunits and led to large steric hindrance to avoid intermolecular *π*-π interactions of azo. This probably further influenced the intermolecular *π*-π and metal-metal interactions of the tridentate platinum (II) moiety resulting in ^3^IL emission. So the luminescent switching between monomer and aggregates can be regulated under light irradiation.

## Materials and Methods

### Materials

Dichloro (1,5-cyclooctadiene)platinum (II), 4-dimethylaminopyridine (DMAP) (*E*)-4-(phenyldiazenyl)aniline (*E*)-4-(phenyldiazenyl)phenol, and 1-ethyl-3-(3-dimethylaminopropyl) carbodiimide hydrochloride (EDC) were purchased from Sigma-Aldrich Chemical Co. 1-Bromobutane, succinic anhydride, 4-ethynylaniline, and prop-2-yn-1-amine were purchased from Aladdin. Unless otherwise indicated, all reagents and materials were obtained from commercial suppliers and were used without purification. The chloroplatinum (II) complex ([Pt (N5C4)Cl]OTf) was synthesized by a modified procedure according to a previously reported method (Li et al., 2013).

### Physical Measurements and Instrumentation

^1^H NMR spectra were recorded on a Bruker DPX 400 FT-NMR spectrometer (400 MHz) at 298 K. Positive ion FAB mass spectra were recorded on a Thermo Scientific DFS high resolution magnetic sector mass spectrometer. Elemental analyses of the complexes were performed on a Flash EA 1112 elemental analyzer. Electronic absorption spectra were obtained using a Varian Cary 50 UV-vis spectrophotometer. Photoirradiation was carried out using a 300 W Xe (ozone-free) lamp. Steady-state excitation and emission spectra at room temperature were recorded on an Edinburgh FS-5 fluorescence spectrofluorometer equipped with a Hamamatsu R-928 P photomultiplier tube. All solutions for the photoisomer studies were deaerated with N_2_ for 15 min. Photophysical measurements at 77 K were carried out with the sample loaded in a quartz tube inside a quartz-walled Dewar flask. Liquid nitrogen was placed into the Dewar flask for low temperature (77 K) photophysical measurements. SEM experiments were performed using a Leo 1530 FEG operating at 4.0–6.0 kV. The SEM sample was prepared by dropping the solution onto a silicon wafer. DLS measurements were performed using an EliteSizer Omni with internal HeNe laser (λ_0_ = 658 nm) from Brookhaven.

### Syntheses

**Syntheses of C1.** A solution of succinic anhydride (200 mg, 2.0 mmol) and a catalytic amount of 4-dimethylaminopyridine (DMAP) in CHCl_3_ (50 ml) was added to a solution of (*E*)-4-(phenyldiazenyl)aniline (394 mg, 2.0 mmol) in CHCl_3_ (50 ml). The reaction mixture was stirred under reflux for 3 h. The solvent was removed under reduced pressure to afford the crude product, which was further purified by column chromatography on silica gel using dichloromethane-acetone (1:1 v/v) in the presence of a few drops of HOAc as eluent to give the desired product. Yield: 518 mg, 87%. ^1^H NMR (400 MHz [D_6_]DMSO, 298 K, TMS): *δ* = 12.16 (br, 1H; -COOH), 10.35 (s, 1H; -CONH-), 7.80–7.89 (m, 6H; -Ph-), 7.51–7.60 (m, 3H; -Ph-), 2.62 (t, *J* = 6.6 Hz, 2H; -COCH_2_-), 2.55 ppm (t, *J* = 6.6 Hz, 2H; -COCH_2_-).

**Syntheses of C2.** The procedure was similar to that for **C1**, except (*E*)-4-(phenyldiazenyl)phenol (394 mg, 2.0 mmol) was used in place of (*E*)-4-(phenyldiazenyl)aniline. The solvent was removed under reduced pressure to afford the crude product, which was further purified by column chromatography on silica gel using dichloromethane-acetone (2:1 v/v) in the presence of a few drops of HOAc as eluent to give the desired product. Yield: 496 mg, 83%. ^1^H NMR (400 MHz [D_6_]DMSO, 298 K, TMS): *δ* = 12.15 (br, 1H; -COOH), 7.76–7.88 (m, 4H; -Ph-), 7.49–7.56 (m, 5H; -Ph-), 2.67 (t, *J* = 6.4 Hz, 2H; -COCH_2_-), 2.56 ppm (t, *J* = 6.4 Hz, 2H; -COCH_2_-).

**Syntheses of L1.** A solution of **C1** (197 mg, 1.0 mmol) and 4-ethynylaniline (117 mg, 1.0 mmol) in dry dichloromethane (100 ml) was added to a solution of 1-ethyl-3-(3-dimethylaminopropyl) carbodiimide hydrochloride (EDC, 384 mg, 2.0 mmol) and a catalytic amount of DMAP in dry dichloromethane (40 ml) under N_2_ and the reaction mixture was stirred at room temperature overnight. Subsequently, the reaction mixture was washed with deionized water and the solvent was removed under reduced pressure. The crude product was further purified by column chromatography on silica gel using dichloromethane-ethyl acetate (2:1 v/v) as eluent to give the desired product. Yield: 237 mg, 80%. ^1^H NMR (400 MHz [D_6_]DMSO, 298 K, TMS): *δ* = 10.38 (s, 1H; -CONH-), 10.19 (s, 1H; -CONH-), 7.80–7.89 (m, 6H; -Ph-), 7.51–7.63 (m, 5H; -Ph-), 7.40 (d, *J* = 9.6 Hz, 2H; -Ph-), 4.06 (s, 1H; -C≡CH), 2.63 (t, *J* = 6.3 Hz, 2H; -COCH_2_-), 2.58 ppm (t, *J* = 6.3 Hz, 2H; -COCH_2_-).

**Syntheses of L2.** The procedure was similar to that for **L1**, except **C2** (197 mg, 1.0 mmol) was used in place of **C1**. The crude product was purified by column chromatography on silica gel using dichloromethane-ethyl acetate (3:1 v/v) as eluent to give the desired product. Yield: 252 mg, 85%. ^1^H NMR (400 MHz [D_6_]DMSO, 298 K, TMS): *δ* = 10.35 (s, 1H; -CONH-), 7.79–7.90 (m, 4H; -Ph-), 7.55–7.64 (m, 7H; -Ph-), 7.48 (d, *J* = 9.2 Hz, 2H; -Ph-), 4.03 (s, 1H; -C≡CH), 2.64 (t, *J* = 6.0 Hz, 2H; -COCH_2_-), 2.55 ppm (t, *J* = 6.0 Hz, 2H; -COCH_2_-).

**Syntheses of L3.** The procedure was similar to that for **L1**, except prop-2-yn-1-amine (55 mg, 1.0 mmol) was used in place of 4-ethynylaniline. Subsequently, the reaction mixture was washed with deionized water and the solvent was removed under reduced pressure. The crude product was purified by column chromatography on silica gel using dichloromethane-ethyl acetate (2:1 v/v) as eluent to give the desired product. Yield: 186 mg, 79% (400 MHz, [D_6_]DMSO, 298 K, TMS): *δ* = 7.87–7.92 (m, 6H; -Ph-), 7.44 (s, 1H, -NH-), 7.49–7.60 (m, 3H; -Ph-), 6.83 (t, *J* = 5.0 Hz, 1H; -CONH-), 3.98 (t, 2H, *J* = 5.3 Hz, -CH_2_N-), 3.17 (t, 1H; *J* = 4.2 Hz, -C≡CH), 2.62 (t, *J* = 6.3 Hz, 2H; -COCH_2_-), 2.58 ppm (t, *J* = 6.3 Hz, 2H; -COCH_2_-).

**Syntheses of L4.** The procedure was similar to that for **L1**, except prop-2-yn-1-ol (56 mg, 1.0 mmol) was used in place of 4-ethynylaniline. Subsequently, the reaction mixture was washed with deionized water and the solvent was removed under reduced pressure. The crude product was purified by column chromatography on silica gel using dichloromethane-ethyl acetate (2:1 v/v) as eluent to give the desired product. Yield: 168 mg, 71%. ^1^H NMR (400 MHz [D_6_]DMSO, 298 K, TMS): *δ* = 7.85–7.91 (m, 4H; -Ph-), 7.50–7.59 (m, 5H; -Ph-), 6.81 (t, *J* = 6.5 Hz, 1H; -CONH-), 3.94 (d, 2H, *J* = 5.4 Hz, -CH_2_N-), 3.15 (t, 1H; *J* = 5.0 Hz, -C≡CH), 2.64 (t, *J* = 6.2 Hz, 2H; -COCH_2_-), 2.57 ppm (t, *J* = 6.2 Hz, 2H; -COCH_2_-).

**Syntheses of 1.** A degassed solution of [Pt (N5C4)Cl]OTf (150 mg, 0.21 mmol) in dichloromethane (30 ml) was added to NEt_3_ (2 ml), a catalytic amount of CuI (2 mg), and **L1** (166 mg, 0.42 mmol). The above solution was stirred at room temperature overnight under N_2_. After the removal of solvents, the residue was purified by column chromatography on silica gel using dichloromethane-acetone (1:1 v/v) as the eluent to give the desired product as a yellow solid. The product was further purified by recrystallization with methanol and dichloromethane. Yield: 140 mg, 63%. ^1^H NMR (400 MHz [D_6_]DMSO, 298 K, TMS): *δ* = 9.31 (s, 1H; -CONH-), 9.07 (s, 1H; -CONH-), 8.78 (t, *J* = 8.2 Hz, 1H, pyridine), 8.45–8.50 (m, 6H; -Ph-), 8.42 (d, *J* = 2.8 Hz, 2H; pyrazole), 8.34 (d, *J* = 9.2 Hz, 2H; -Ph-), 8.07–8.14 (m, 5H; -Ph-), 7.85 (d, *J* = 8.2 Hz, 2H; pyridine), 7.63 (d, *J* = 2.8 Hz, 2H; pyrazole), 5.42 (t, *J* = 7.2 Hz, 4H; -NCH_2_-), 2.63 (t, *J* = 6.3 Hz, 2H; -COCH_2_-), 2.58 (t, *J* = 6.3 Hz, 2H; -COCH_2_-), 1.87–1.93 (m, 4H; -CH_2_-), 1.42–1.46 (m, 4H; -CH_2_-), 1.05 ppm (t, *J* = 7.3 Hz, 6H; -CH_3_). ^13^C NMR (100 MHz [D_6_]DMSO, 298 K, TMS): *δ* = 171.8, 171.3, 152.6, 150.3, 148.3, 147.5, 143.1, 137.2, 132.1, 131.3, 129.6, 124.0, 122.3, 120.1, 118.5, 108.6, 88.5, 70.0, 52.6, 35.2, 27.3, 19.5, 14.3 ppm. Positive FAB−MS: m/z 913 [M−OTf]^+^. Anal. Calcd (%) for C_44_H_44_F_3_N_9_O_5_PtS: C, 49.71; H, 4.17; N, 11.86; found: C, 49.53; H, 4.38; N, 11.59.

**Syntheses of 2.** The procedure was similar to that for **1**, except **L2** (166 mg, 0.42 mmol) was used in place of **L1**. After the removal of solvents, the residue was purified by column chromatography on silica gel using dichloromethane-acetone (1:1 v/v) as the eluent to give the desired product as a yellow solid. The product was further purified by recrystallization with methanol and dichloromethane. Yield: 128 mg, 57%. ^1^H NMR (400 MHz [D_6_]DMSO, 298 K, TMS): *δ* = 9.04 (s, 1H; -CONH-), 8.78 (t, *J* = 8.5 Hz, 1H, pyridine), 8.46–8.52 (m, 4H; -Ph-), 8.42 (d, *J* = 2.6 Hz, 2H; pyrazole), 8.32 (d, *J* = 9.1 Hz, 2H; -Ph-), 8.02–8.12 (m, 7H; -Ph-), 7.83 (d, *J* = 8.5 Hz, 2H; pyridine), 7.61 (d, *J* = 2.6 Hz, 2H; pyrazole), 5.42 (t, *J* = 7.0 Hz, 4H; -NCH_2_-), 2.62 (t, *J* = 6.2 Hz, 2H; -COCH_2_-), 2.56 (t, *J* = 6.2 Hz, 2H; -COCH_2_-), 1.85–1.92 (m, 4H; -CH_2_-), 1.41–1.45 (m, 4H; -CH_2_-), 1.01 ppm (t, *J* = 6.8 Hz, 6H; -CH_3_). ^13^C NMR (100 MHz [D_6_]DMSO, 298 K, TMS): *δ* = 172.0, 171.4, 155.4, 152.4, 149.5, 147.6, 142.9, 137.0, 132.3, 131.8, 129.9, 124.1, 122.8, 120.4, 119.6, 108.3, 89.2, 70.4, 53.4, 32.7, 27.5, 19.4, 14.0 ppm. Positive FAB−MS: m/z 914 [M−OTf]^+^. Anal. Calcd (%) for C_44_H_43_F_3_N_8_O_6_PtS: C, 49.67; H, 4.07; N, 10.53; found: C, 49.58; H, 4.40; N, 10.37.

**Syntheses of 3.** The procedure was similar to that for **1**, except **L3** (140 mg, 0.42 mmol) was used in place of **L1**. After the removal of solvents, the residue was purified by column chromatography on silica gel using dichloromethane-acetone (1:1 v/v) as the eluent to give the desired product as a yellow solid. The product was further purified by recrystallization with methanol and dichloromethane. Yield: 96 mg, 46%. ^1^H NMR (400 MHz [D_6_]DMSO, 298 K, TMS): *δ* = 8.80 (t, *J* = 8.6 Hz, 1H, pyridine), 8.43 (d, *J* = 2.6 Hz, 2H; pyrazole), 8.33–8.39 (m, 7H; -Ph- and -CONH-), 8.01–8.11 (m, 3H; -Ph-), 7.85 (d, *J* = 8.6 Hz, 2H; pyridine), 7.63 (d, *J* = 2.6 Hz, 2H; pyrazole), 7.06 (t, *J* = 5.1 Hz, 1H; -CONH-), 5.43 (t, *J* = 6.8 Hz, 4H; -NCH_2_-), 4.56 (d, 2H, *J* = 5.1 Hz, -CH_2_N-), 2.64 (t, *J* = 6.1 Hz, 2H; -COCH_2_-), 2.57 (t, *J* = 6.1 Hz, 2H; -COCH_2_-), 1.83–1.94 (m, 4H; -CH_2_-), 1.40–1.46 (m, 4H; -CH_2_-), 1.01 ppm (t, *J* = 6.8 Hz, 6H; -CH_3_). ^13^C NMR (100 MHz [D_6_]DMSO, 298 K, TMS): *δ* = 172.2, 171.8, 153.1, 151.0, 148.7, 147.7, 143.5, 131.8, 131.0, 124.3, 118.7, 108.5, 88.9, 70.6, 52.7, 35.2, 29.9, 27.3, 19.3, 14.5 ppm. Positive FAB−MS: m/z 851 [M−OTf]^+^. Anal. Calcd (%) for C_39_H_42_F_3_N_9_O_5_PtS: C, 46.80; H, 4.23; N, 12.59; found: C, 46.43; H, 4.51; N, 12.46.

**Syntheses of 4.** The procedure was similar to that for **1**, except **L4** (140 mg, 0.42 mmol) was used in place of **L1**. After the removal of solvents, the residue was purified by column chromatography on silica gel using dichloromethane-acetone (1:1 v/v) as the eluent to give the desired product as a yellow solid. The product was further purified by recrystallization with methanol and dichloromethane. Yield: 105 mg, 50%. ^1^H NMR (400 MHz [D_6_]DMSO, 298 K, TMS): *δ* = 8.82 (t, *J* = 8.6 Hz, 1H, pyridine), 8.46 (d, *J* = 2.6 Hz, 2H; pyrazole), 8.35–8.40 (m, 4H; -Ph-), 8.03–8.12 (m, 5H; -Ph-), 7.86 (d, *J* = 8.6 Hz, 2H; pyridine), 7.65 (d, *J* = 2.6 Hz, 2H; pyrazole), 7.07 (t, *J* = 5.0 Hz, 1H; -CONH-), 5.43 (t, *J* = 6.7 Hz, 4H; -NCH_2_-), 4.54 (d, 2H, *J* = 5.0 Hz, -CH_2_N-), 2.65 (t, *J* = 6.0 Hz, 2H; -COCH_2_-), 2.57 (t, *J* = 6.0 Hz, 2H; -COCH_2_-), 1.84–1.92 (m, 4H; -CH_2_-), 1.43–1.48 (m, 4H; -CH_2_-), 1.03 ppm (t, *J* = 6.8 Hz, 6H; -CH_3_). ^13^C NMR (100 MHz [D_6_]DMSO, 298 K, TMS): *δ* = 172.2, 171.6, 154.4, 152.8, 149.2, 147.6, 143.3, 131.8, 124.5, 119.6, 108.8, 88.2, 70.4, 52.8, 34.6, 30.2, 27.5, 19.8, 14.3 ppm. Positive FAB−MS: m/z 852 [M−OTf]^+^. Anal. Calcd (%) for C_39_H_41_F_3_N_8_O_6_PtS: C, 46.75; H, 4.12; N, 11.18; found: C, 46.55; H, 4.23; N, 11.52.

## Conclusion

In conclusion, we designed and synthesized four azo-appended alkynylplatinum (II) complexes with precisely modified amide or ester linkers. The different linkers endowed these complexes with discrepancies in self-assembly, and photophysical and photoisomerization properties because of the formation of intermolecular hydrogen bonding, *π*-π stacking, and metal-metal interactions. The complexes exhibited emission bands which originated from ^3^IL or ^3^MMLCT excited states depending on the assembly properties and *cis* and *trans* isomers. The morphologies could also be modulated between nanofibers and nanospheres. So the regulation of switchable luminescent properties and aggregated morphologies can be realized by photoisomerization of the azo moiety under UV and visible light irradiations. This also provided us with a convenient method to identify the assembly behaviors of luminescent materials by monitoring the emission or color changes.

## Data Availability Statement

The raw data supporting the conclusions of this article will be made available by the authors, without undue reservation.

## Author Contributions

The majority of the experiments and data analysis were carried out by YL. All the authors discussed the results. The manuscript was prepared and revised by YL, YF, HS, SY, and JL.

## Funding

This work was supported by the National Natural Science Foundation of China (Grant Nos. 21871297, 21503284, and 22001054), the National Key R&D Program of China (Grant Nos. 2020YFA0908503 and No. 2018YFA0901600), the Hangzhou Overseas High-level Talent (Teams) Innovation and Entrepreneurship Program (Grant No. 4095C5062000604), the Climbing Plan Project of Hangzhou Normal University (Grant No. 4095C5021910201), and the support from Hangzhou Normal University.

## Conflict of Interest

The authors declare that the research was conducted in the absence of any commercial or financial relationships that could be construed as a potential conflict of interest.
